# Continuous Shoulder Activity Tracking after Open Reduction and Internal Fixation of Proximal Humerus Fractures

**DOI:** 10.3390/bioengineering10020128

**Published:** 2023-01-18

**Authors:** Michiel Herteleer, Armin Runer, Magdalena Remppis, Jonas Brouwers, Friedemann Schneider, Vasiliki C. Panagiotopoulou, Bernd Grimm, Clemens Hengg, Rohit Arora, Stefaan Nijs, Peter Varga

**Affiliations:** 1 Department of Trauma Surgery, University Hospitals Leuven, 3000 Leuven, Belgium; 2 University Hospital for Orthopaedics and Traumatology, Medical University Innsbruck, 6020 Innsbruck, Austria; 3 Orthopaedic Sports Medicine, Klinikum Rechts der Isar, Technical University of Munich, 80333 Munich, Germany; 4 AO Research Institute Davos, 7270 Davos, Switzerland; 5 Department of Precision Health, Luxembourg Institute of Health, 1445 Strassen, Luxembourg; 6 Utrecht Medical Centre Utrecht, 3584 CX Utrecht, The Netherlands

**Keywords:** shoulder activity, sensor, rehabilitation protocol, proximal humerus fracture

## Abstract

Postoperative shoulder activity after proximal humerus fracture treatment could influence the outcomes of osteosynthesis and may depend on the rehabilitation protocol. This multi-centric prospective study aimed at evaluating the feasibility of continuous shoulder activity monitoring over the first six postoperative weeks, investigating potential differences between two different rehabilitation protocols. Shoulder activity was assessed with pairs of accelerometer-based trackers during the first six postoperative weeks in thirteen elderly patients having a complex proximal humerus fracture treated with a locking plate. Shoulder angles and elevation events were evaluated over time and compared between the two centers utilizing different standard rehabilitation protocols. The overall mean shoulder angle ranged from 11° to 23°, and the number of daily elevation events was between 547 and 5756. Average angles showed longitudinal change <5° over 31 ± 10 days. The number of events increased by 300% on average. Results of the two clinics exhibited no characteristic differences for shoulder angle, but the number of events increased only for the site utilizing immediate mobilization. In addition to considerable inter-patient variation, not the mean shoulder angle but the number of elevations events increased markedly over time. Differences between the two sites in number of daily events may be associated with the different rehabilitation protocols.

## 1. Introduction

Proximal humeral fractures are common fractures in the elderly and affecting up to 111 per 100,000 persons per year [1]. In displaced three- or four-part fractures, open reduction and internal fixation (ORIF) aims at the best possible restoration of shoulder anatomy and thus shoulder function [2,3,4]. Shoulder function after ORIF mainly improves between 3 and 12 months after surgery but acute loss of reduction usually happens within 6 weeks after surgery [5,6,7,8]. In addition to other factors such as the donor’s age and sex, bone stock quality, complexity and reduction quality of the fracture, comorbidities, fixation type and augmentation, the rehabilitation protocol may contribute to these early failures [9,10,11,12,13,14,15,16,17,18]. There are differences in rehabilitation programs after ORIF whereas some surgeons stimulate an immediate functional non-weight bearing rehabilitation program, while others have a less aggressive approach and prefer an initial physiotherapist assisted rehabilitation program [19]. It remains unclear what impact these different rehabilitation programs have on postoperative patient satisfaction, return to function, complications and failures [20]. Moreover, it remains challenging to capture the frequency and extent of shoulder activity performed by a patient throughout the day. Technological advancements allow recording patient activity via trackers and motion capture sensors, allowing continuous assessment of activities of daily life for periods ranging from a few days up to several weeks [21,22].

The goal of this pilot study was threefold. The first aim was to evaluate the feasibility of continuously monitoring shoulder activity over a period of several weeks. The second aim was to describe the evolution of shoulder activity within the first six postoperative weeks in proximal humerus fracture patients treated with locked plate osteosynthesis. The third aim was to evaluate potential differences in the degree of postoperative shoulder activity between two different rehabilitation protocols.

## 2. Materials and Methods

This multi-centric prospective study investigated shoulder activity with accelerometer-based trackers during the first six postoperative weeks in elderly patients with a complex proximal humerus fracture treated with the PHILOS plate (DePuy Synthes, Zuchwil, Switzerland). The two study centers were the University Hospitals Leuven and Medical University Innsbruck. Note that the study sites will be referred to in an anonymized manner below. The study was approved by the local ethical committees (approval numbers S62376 and 1281/2018, respectively).

### 2.1. Patient Recruitment

Inclusion criteria were age ≥ 50 years, displaced or unstable three- or four-part fracture of the proximal humerus (except isolated displaced fractures of the greater or lesser tuberosity) treated with a plate and screw osteosynthesis (PHILOS locking plate—with or without screw augmentation) within 10 days after injury, ability to understand the content of the study and the patient consent form and voluntary signed informed consent.

Exclusion criteria were previous proximal humerus fracture on the ipsilateral limb, humeral head impression/splitting fracture, fibula grafting, bone block or any other non-cement augmentation of the PHILOS locking plate fixation, associated nerve or vessel injury, serious fracture fixation issues such as too long screw, screw perforation through the humeral head, or a broken screw or implant recognized directly on the first postoperative X-ray. Other exclusion criteria were severe systematic diseases rated in class 4 and higher of the American Society of Anesthesiologists (ASA) physical status classification, substance abuse, prisoner, participation in another medical device or product study in the past month that could affect this study, pregnancy, or pacemaker.

Patient data including age, gender, height, weight, residential status, injury side, arm dominance and fracture type were collected at recruitment.

### 2.2. Postoperative Protocol

The two university hospitals used different postoperative rehabilitation protocols according to their standard of care. In hospital H1, the patients were treated with a sling for 3 weeks and were only allowed passive and active-assisted mobilization under supervision of a physiotherapist for the first 3 weeks. Patients in hospital H2 were treated without a sling and allowed to mobilize without restrictions immediately. Physiotherapy was started immediately postoperatively and prescribed 2–3 times per week in both hospitals.

### 2.3. Activity Tracking Apparatus and Procedure

**Figure 1:**
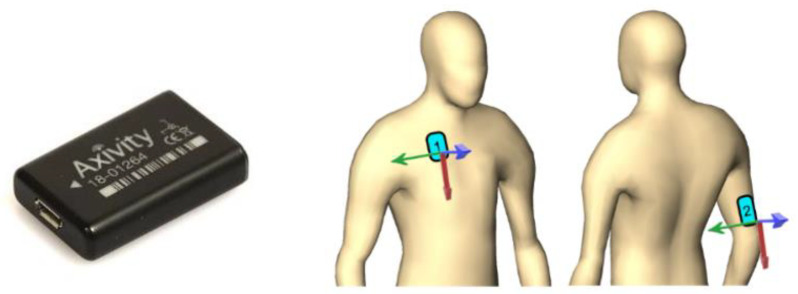
Accelerometer sensors (Axivity AX3, (**left**), source: https://axivity.com/) were attached to the patients (illustrated on the (**right**)), on the chest (“1”) and on the back of the upper arm on the treated side (“2”). Each sensor had its own coordinate system, indicated with the red–green–blue arrows.

Accelerometer sensors (AX3, Axivity Ltd., Newcastle upon Tyne, UK) [23] (Figure 1, left) were used to measure shoulder activity continuously (24/7) for 6 weeks after the operation in two consecutive 3-week periods. The length of the measurement period was determined by the sensor’s battery and memory capacities. The first period started at the latest 4 days postoperatively and ended at 21 ± 3 days, the second period started at the same visit and ended at 42 ± 3 days. Two sensors were used for each patient and period. One sensor was attached to the upper arm of the treated side, and another was located at the chest and served as a reference (Figure 1, right), allowing evaluation of the shoulder angle as the orientation difference between both devices. Data recording was performed at 50 Hz frequency within ±4 g limits that were deemed suitable in a pilot evaluation. The sensors were attached to the skin using a dedicated certified medical-grade adhesive tape (3M 4077, 3M Medical Materials & Technologies, Oakdale, CA, USA). Attachment (directly postoperatively and at the 3-week follow-up visit) and detachment (at the 3-week and 6-week follow-up visits) were performed by trained study personnel according to standard operating procedures to ensure consistent sensor location and alignment. The start and end time points of a given period were marked by knocking five times synchronously at both arm and chest sensors. The patients were allowed to follow their normal daily activities including showering with the attached device. At the end of the measurement periods and after detachment, sensor data were downloaded using the Open Movement GUI software (Open Movement project).

### 2.4. Data Processing

The raw data of the sensors were corrected, filtered, synchronized, and evaluated using Matlab (R2020b, MathWorks, Natick, MA, USA) as follows. Calibration was performed to compensate for imperfections in magnitude and directional errors of each sensor such that the acceleration was measured in stationary position equal to 1.0 g (gravity, i.e., 9.81 m/s^2^ acceleration) in each direction, and the orientation of the measured vector is perpendicular to the planar sides of the sensor’s housing when resting on a horizontal flat surface [24]. The detailed description of these calibrations is provided in Appendix A. The quantified imperfections were used to correct the raw data.

Since the arm and chest sensors were recording independently, their data needed to be synchronized in time. This required shifting and scaling operations based on the time landmarks defined by the knocking events at attachment and detachment. Shifting was achieved based on the starting point of the activity assessment, which was dictated by five knocking events on both sensors directly after attachment. Scaling was evaluated and corrected based on the lengths of the measurement periods of the two sensors determined by the time difference of the initial and final knocking events; interpolation ensured that the data were available at the same time points for both sensors.

The measurement noise of the raw data was alleviated using a combination of a low-pass Butterworth filter with a cut-off of 5 Hz to remove high-frequency noise, which was followed by a smoothing step using a moving average filter utilizing quadratic regression with a window size of 60 ms.

Shoulder angle was calculated as the rotational difference between the coordinate systems of the arm sensor versus the chest sensor (Figure 1, right). The calculation method was validated in an experimental setup to ensure <2° accuracy in the angles between the two sensors. The 0° angle was defined at the initial knocking event performed directly after sensor attachment, at neutral position of the shoulder with the patient being in an upright position. Due to the limitation that accelerometer sensors can determine their orientation only with respect to the gravity vector in steady states, it was not possible to discriminate the different anatomical components of shoulder rotation. Thus, only a single shoulder angle integrating components of flexion–extension and adduction–abduction could be determined. Large acceleration events, i.e., >1.5 g, were excluded to ensure reliable orientation assessment for the sensors. Moreover, the part of the data related to the sleeping and resting periods of the patients was excluded, as these periods were not of primary interest and could not be reliably assessed due to the technical limitations of accelerometers and their attachment to the skin. Therefore, the final evaluation was restricted to periods when the upper body posture was between −30° and +30°, as assessed by the chest sensor.

Shoulder elevations, referred to below as “events”, were determined as peaks between increasing and subsequent decreasing angle in the shoulder activity data with a minimum prominence of 10°.

The average magnitude of shoulder angles and number of shoulder elevation events were evaluated for each post-operative day. Additionally, the changes compared to the direct postoperative status; i.e., the average of the 2–5 postoperative days were evaluated for both the average shoulder angle and number of elevation events. These relative values allowed for a more direct comparison between patients. The two different rehabilitation protocols were compared by averaging the data of all patients per hospital and comparing the outcomes of the hospitals.

## 3. Results

This feasibility study included 14 patients (11 women and 3 men), with seven patients treated in each hospital H1 and H2 (Table 1). One patient from hospital H2 had to be excluded from follow-up because of discomfort wearing the activity trackers. Mean age at time of surgery for the remaining thirteen patients was 63 ± 8 years. Five patients were living alone. The injured and tracked arm was the dominant arm in eight patients. Sling removal time was 23 ± 4.5 days and 2 ± 1.5 days in hospitals H1 and H2, respectively.

The total recording time was on average 31 ± 10 days (mean ± standard deviation (SD)). Ten patients had measurements in both first and second 3-weeks periods. Two patients had mild adverse event in form of skin irritation or reactions at the sensor attachment site during the first recording period and could not complete the second period. All adverse events were fully resolved by three months. Another four patients experienced mildly irritated, red skin but were able to take part in the second recording period.

The tracker of four patients ran out of battery before the end of the measurement period, and thus, the collected data were not complete. A single sensor broke and did not allow the data to be accessed. Six patients reported incidents of tape detachment; these were reattached by the patients themselves. Wherever possible, the date and time of de- and reattachments were assessed by the patient and the study personnel. Analysis of the sensor data allowed to identify and correct or exclude these parts of the data.

**Figure 2:**
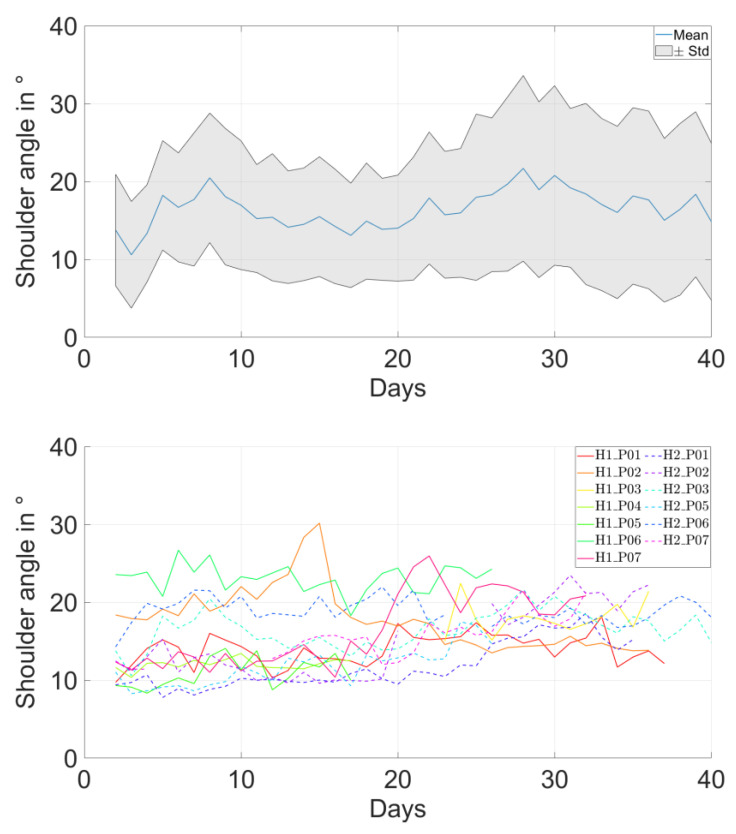

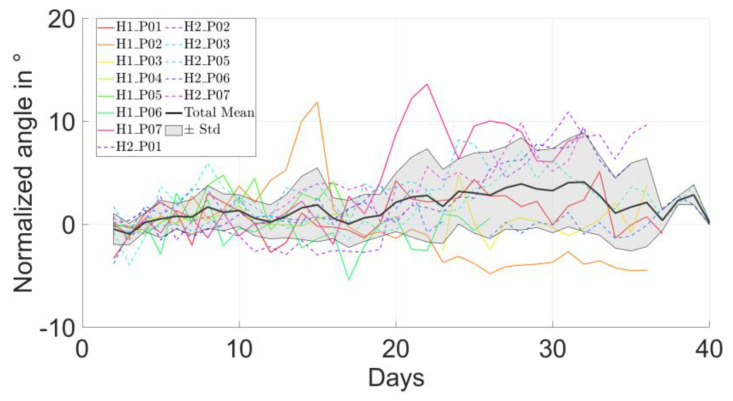
Daily average shoulder angle results. (**Top**): the mean (blue line) and standard deviation (gray zone) of shoulder angle for each postoperative day of a patient (H2_P04). The overall mean ± standard deviation of shoulder angle for the entire tracking period of this patient was 16.3 ± 9.3°. (**Middle**): absolute daily average of the shoulder angles for each patient. (**Bottom**): normalized (compared to the direct postoperative state) daily average of the shoulder angles for each patient. Note that the data were not available throughout the entire six-week period for some patients.

The overall mean shoulder angle ranged between 11° and 23° in all patients (Table 1). The evolution of daily average of shoulder angle over time showed no longitudinal change for most patients (Figure 2 top and middle). This trend was confirmed by the evolution of the relative change of shoulder angle compared to the direct postoperative days, remaining smaller than 5° (Figure 2, bottom).

**Figure 3:**
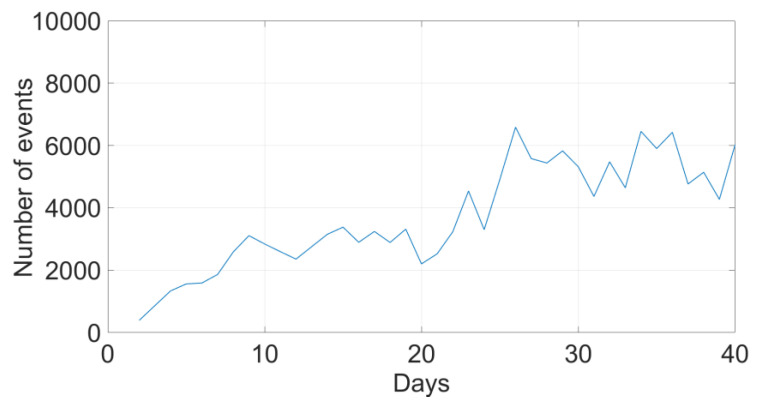

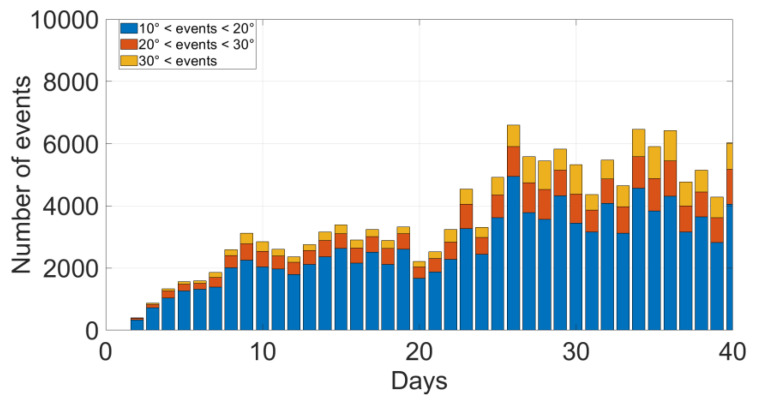
Number of shoulder elevation events of one patient (H2_P04). (**Top**): evolution of the number of shoulder elevation events throughout the tracking period. (**Bottom**): evolution of the number of shoulder elevation events throughout the tracking period categorized into three ranges according to the maximum shoulder angle reached. The daily mean ± standard deviation of shoulder elevation events of this patient was 4109 ± 1684.

**Figure 4:**
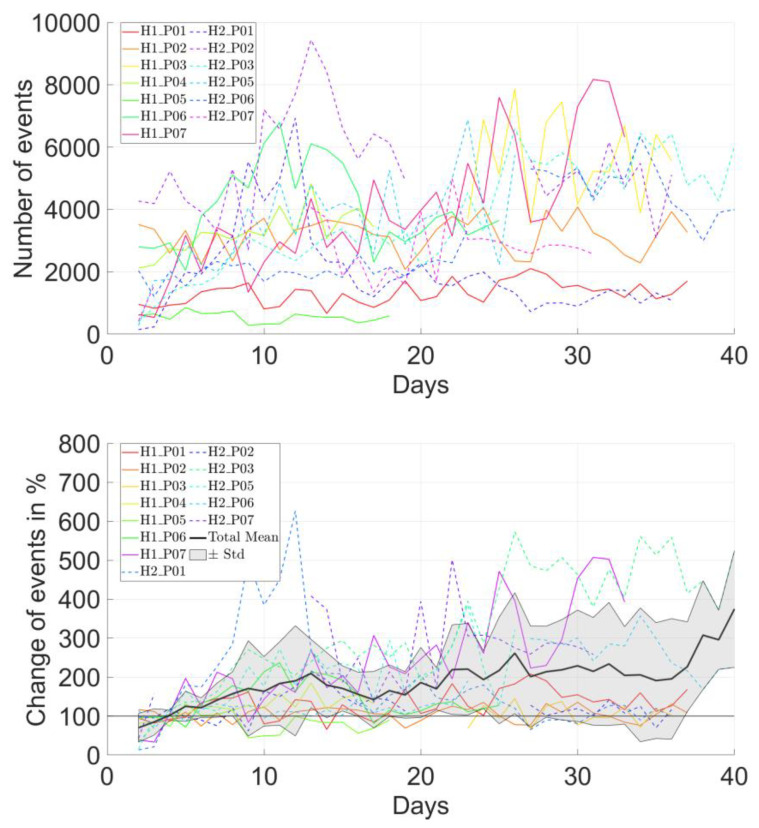
Absolute (**top**) and relative ((**bottom**), compared to the direct postoperative state) number of daily shoulder elevations larger than 10°, shown for each patient. Note that the data were not available throughout the entire six-week period for some patients.

The overall average number of daily shoulder elevation events ranged between 547 and 5756 in all patients (Table 1). The number of daily events increased for most but not all patients (Figure 3 and Figure 4, top). The relative change of daily event numbers compared to the direct postoperative days showed an increasing trend over time, reaching up to 300% increase (Figure 4, bottom).

**Figure 5:**
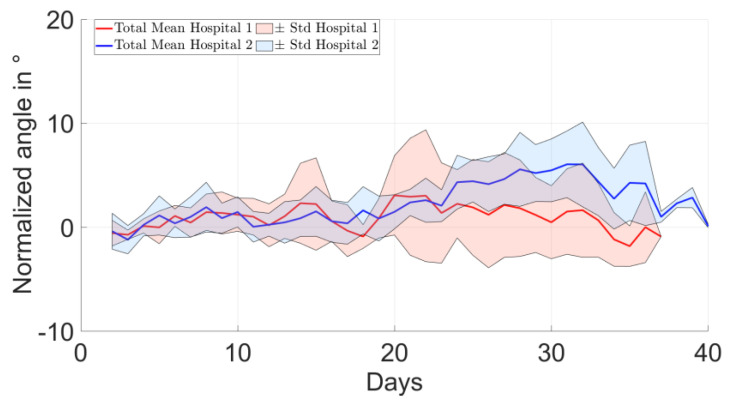
Comparison of the average results of the two clinical sites, i.e., rehabilitation protocols, in terms of the change in the shoulder angle compared to the direct postoperative period.

**Figure 6:**
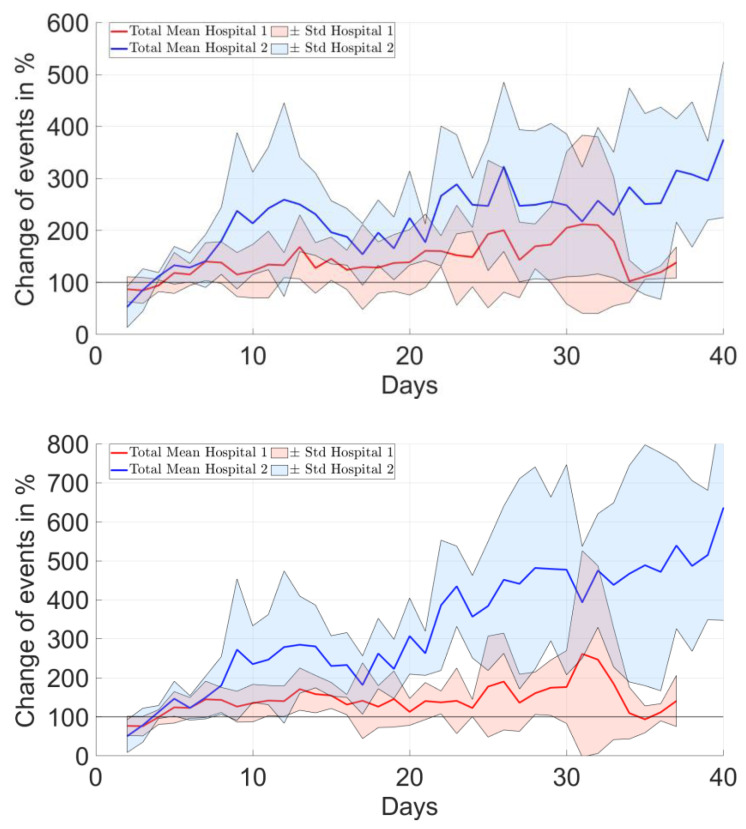

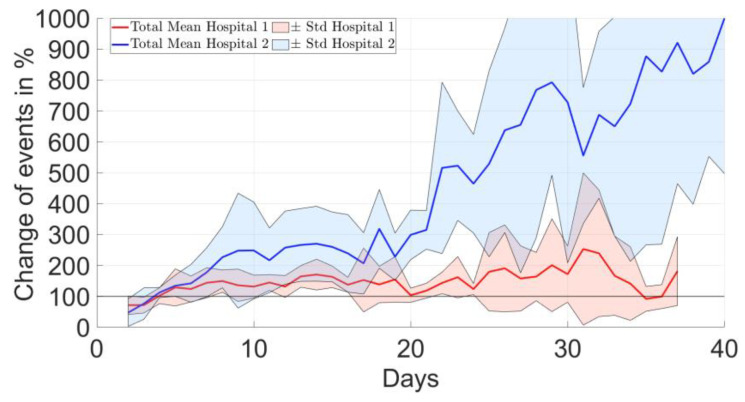
Comparison of the average results of the two clinical sites, i.e., rehabilitation protocols, in terms of the change in the total number of shoulder elevation events over time. The sub-figures show the data for shoulder elevation events beyond 10° (**top**), 20° (**middle**) and 30° (**bottom**).

There were no characteristic differences between the two clinical sites, i.e., rehabilitation protocols, in terms of the longitudinal evolution of the change in the average shoulder angle (Figure 5). However, the evolution of the percentile changes in the number of events relative to the postoperative period was increasing for H2 but not for H1, and the differences between sites became more pronounced for higher elevation thresholds (Figure 6).

## 4. Discussion

The primary findings of this study were that in patients after proximal humerus fracture undergoing ORIF, the mean shoulder angle varied up to a factor 2 between individuals, but it hardly increases in the first six weeks. The number of events exhibited a 10-fold difference between subjects, and the time evolution of event numbers showed an increasing trend. The comparison of the two hospitals indicated that the rehabilitation protocol might affect the number of daily shoulder elevation events with patients following an immediate functional non-weight bearing rehabilitation program having a higher number of events, especially for large shoulder angles.

Postoperative rehabilitation protocols after proximal humerus ORIF vary substantially between different hospitals and surgical centers ranging from strict immobilization using a shoulder sling to a more progressive, functional non-weight-bearing approach without sling immobilization [25]. If immobilized, the duration of postoperative sling usage ranged from none to eight weeks [25]. A recent review summarizing five comparative studies did not find any benefit of longer sling immobilization compared to early functional therapy. While exercise and early functional mobilization is clearly advised, the amount and influence of postoperative mobilization and its effect on clinical and subjective outcomes as well as revision and failure rates are still unknown [26,27].

When recording physical activity or joint motion, wearable activity trackers are frequently used, and single day data acquisition was our method of choice [28]. While this might be less burdensome for the patient in comparison to long-term recording, the informative value of these data is limited and long-term recordings are recommended. To the best of the authors’ knowledge, the present work represents the first study providing detailed insights into the longitudinal evolution of postoperative shoulder activity of surgically treated patients with proximal humerus fracture. The relevance of long-term measurement was underlined by the time evolution of the assessed parameters. Although the average daily shoulder angles remained fairly constant over time, the number of shoulder angles showed an increasing trend over time, exhibiting important differences between patients, especially after three weeks postoperatively and in particular for large shoulder angle events. These may be related to the different rehabilitation protocols adopted by both investigation sites. While in the first two weeks, there is no difference with regard to the total number of elevation events >10°, an increased number of events for patients following the unrestricted, i.e., non-weight bearing, rehabilitation protocol was recorded thereafter. For non-operatively treated proximal humerus fractures, early active rehabilitation yields equal complications and shoulder functions as prolonged sling immobilization and restricted rehabilitation [26,29]. Similarly, the present data suggest a potential benefit of early active rehabilitation in terms of faster return to motion and function compared to a more restricted rehabilitation protocol. Nevertheless, the influence of a patients’ preoperative activity level on the amount of shoulder activity in the early postoperative phase is still unknown, and therefore, caution is needed when interpreting these results.

Recording detailed postoperative shoulder activity, using wearables is challenging, and little high-quality knowledge exists. Van de Kleut et al. investigated daily shoulder activity before and after reverse Total Shoulder Arthroplasty (rTSA) using Inertial Measurement Units (IMUs) [30]. Their results showed an increased frequency of arm elevations to higher angles but no difference in the amount of time spent in the elevation. Moreover, shoulder elevation accounted for less than 1% of daily shoulder motion, and even after one year postoperatively, patients spent more than 95% of the day in shoulder angles below 60° [30]. These results compare to the present work, where patients spent 94% of the time in shoulder angles below 40°. The initial increase in shoulder events seen in the present study may be due to postoperative physiotherapy, which is in line with previously reported data showing a significant increase in events only in the early postoperative period but not thereafter [30]. This can be explained by the fact that physiotherapy is adapted to the state of the patient starting with simple exercises that become more challenging over time. Therefore, it is more likely to see a general increase in activity over time which is the case in the present work. Furthermore, physiotherapy is performed only during a limited amount of time during the day and might therefore have only a limited effect on daily shoulder activity. In general, the present data show that after open reduction and internal fixation, the shoulder activity level of patients is low and that the early return to full range of motion is not seen in the first weeks.

This study has some limitations. The small number of patients included into this pilot study did not allow for meaningful statistical analysis to be performed, but the indicated trends can be used to design more specific and focused investigations. Moreover, the feasibility of long-term tracking was assessed, providing novel insights and highlighting potential pitfalls. Technical limitations included the issue that the battery of the activity trackers did not always last for the desired time window of three weeks. Since for the calculation of the shoulder angle, the recordings of both activity trackers are necessary, the analysis could only be conducted as long as both activity trackers were functional. Detachment of the tape fixing the sensors occurred due to the loss of adhesion to the skin or ruptures of the material, causing a partial unavailability of data until reattachment. The assessed shoulder angles were not validated against optical tracking techniques. However, the method for calculating the shoulder angle used here was similar to the one applied by Chapman et al. [31], who validated their results against a laboratory motion capture system and reported errors smaller than 2° for abduction, forward flexion, internal and external rotation. In addition, over the long-term, i.e., days to weeks, activity monitoring application with thousands of events, the accuracy of a single event is less critical as the focus is on behavioral change and the large data sample compensates for a potentially lower accuracy compared to what would be needed during a single functional test. Shoulder activity monitoring by counting events beyond certain joint angle thresholds may be affected by the lifestyle of the subject. Thus, correct interpretation of the absolute number of events would require a pre-trauma reference. With the latter being hardly possible, in future studies, the unaffected shoulder could be monitored simultaneously for an intra-subject reference and potential transfer of activities during the rehabilitation phase.

## 5. Conclusions

Activity tracker-based continuous shoulder activity assessment in patients with a complex proximal humerus fracture treated with a locking plate was feasible and revealed that the mean value of the shoulder angle had up to two times differences between individuals but hardly increased during the first 6 postoperative weeks for most patients. Up to 10-fold differences in the daily shoulder elevation events between patients could be seen. There was a considerable difference in the number of shoulder elevation events > 10° between patients of both hospitals, which may be due to different rehabilitation protocols. Event counts above a functionally demanding threshold seemed to be the most sensitive digital mobility parameter monitoring post-traumatic recovery and may streamline wearable sensor data analysis in future studies as well as establish comparability between trials. These observations require confirmation by future studies including a larger cohort. When applied to a larger group of patients, the presented methods could be used in future studies to objectively and functionally evaluate the effect of postoperative activity on the outcomes of proximal humerus fracture fixation and to assess patient compliance. The resulting data could serve as the basis for developing improved and potentially personalized rehabilitation protocols and guidance for the patient.

## Figures and Tables

**Table 1:** Demographic data, shoulder angles and number of daily shoulder elevation events of the patients involved in the study. Sex: F = female and M = male. SD refers to standard deviation.

Patient ID	Age in Years	Sex	Shoulder Angle in °		Number of Daily Events	
			Mean	SD	Mean	SD
H1_P01	79	F	14	6.9	1325	357
H1_P02	60	F	18	9.7	3170	554
H1_P03	52	F	17.9	12.1	5756	1440
H1_P05	58	M	12	7.9	3267	693
H1_P06	72	F	11	8	547	159
H1_P07	58	F	23	9.9	4073	1305
H2_P01	61	F	16.4	4.8	3942	2016
H2_P01	76	F	11.8	4.6	1996	1450
H2_P03	63	F	14.8	6.2	5407	1558
H2_P04	56	F	16.3	9.3	4109	1684
H2_P05	69	F	11.3	7.1	3421	1468
H2_P06	54	M	18.6	9.2	3385	1434
H2_P07	58	M	15.3	8.1	2517	1040

**Figure A1:**
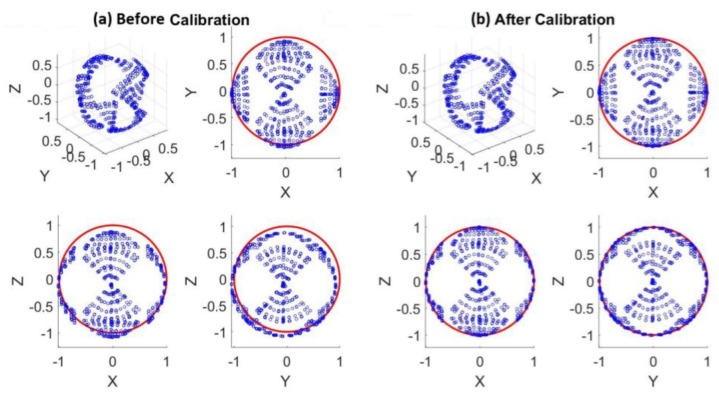
Illustration of the accelerometer’s magnitude calibration. Data points shown in blue represent accelerations in units of gravity measured in different directions for stationary periods, and the red circle shows the target unit sphere with a radius of 1 g. Imperfections before calibration (**a**) are resolved by the corrections after calibration (**b**).

## Data Availability

Not applicable.

## Appendix A. Sensor Data Processing

Magnitude calibration of the activity sensors was performed by calculating a correction factor to rescale the vector magnitude in stationary periods to the value of 1 g (9.81 m/s^2^) in numerous directions. The sensor was mounted to a special vise allowing angulations in two directions, and acceleration data were recorded for 20 s of stationary periods. This was repeated for different orientations covering a sphere with steps on 10°. Imperfections in the offset (eccentricity) and magnitude (scaling) were corrected via iterative closest point fitting of the measured points to the target unit sphere (Figure A1). The resulting correction factors were used to correct the recorded data.

Directional calibration of the sensors aimed to correct for potential obliqueness of the sensor positioning within the housing. Acceleration data were recorded for 10 s of stationary periods while positioning the housing with one of its sides on a horizontal surface. Correction was then applied to match recorded data with the target unit vector of 1 g length for each side.
